# A novel inflammatory signature for evaluating immune microenvironment status in soft tissue sarcoma

**DOI:** 10.3389/fonc.2022.990670

**Published:** 2022-10-13

**Authors:** Zhehong Li, Honghong Zheng, Lirui Liu, Zhen Fen, Haiying Cao, Jilong Yang, Junqiang Wei

**Affiliations:** ^1^ Department of Traumatology and Orthopaedics, Affiliated Hospital of Chengde Medical University, Chengde, China; ^2^ Department of General Surgery, Beijing Shijitan Hospital, Capital Medical University, Beijing, China; ^3^ Department of General Surgery, Affiliated Hospital of Chengde Medical University, Chengde, China; ^4^ Department of Neonatal Department, Affiliated Hospital of Chengde Medical University, Chengde, China; ^5^ Department of Bone and Soft Tissue Tumor, Tianjin Medical University Cancer Institute and Hospital, Tianjin, China; ^6^ National Clinical Research Center for Cancer, Key Laboratory of Cancer Prevention and Therapy, Tianjin’s Clinical Research Center for Cancer, Tianjin, China

**Keywords:** soft tissue sarcoma, inflammatory landscape, tumor microenvironment, immune, cancer

## Abstract

**Background:**

Tumorigenesis and progression are intimately associated with inflammation. However, the inflammatory landscape in soft tissue sarcoma (STS) and its clinical consequences are yet unknown, and more investigation is needed.

**Methods:**

RNA-seq expression data for STS and corresponding normal tissues were downloaded from The Cancer Genome Atlas database and the Genotype-Tissue Expression Portal. Differential and prognostic analyses were performed based on known inflammatory response genes from Gene Set Enrichment Analysis (GSEA). We utilized LASSO-Cox analysis to determine hub genes and built an inflammatory score (INFscore) and risk stratification model. Furthermore, a nomogram, including the risk stratification model, was established to predict the prognosis. We further elucidated the characteristics among different risk STS patients by GSEA, gene set variation analysis, and detailed immune infiltration analysis. Finally, the INFscore and risk stratification model in predicting prognosis and depicting immune microenvironment status were verified by pan-cancer analysis.

**Results:**

Five hub genes (HAS2, IL1R1, NMI, SERPINE1, and TACR1) were identified and were used to develop the INFscore. The risk stratification model distinguished the immune microenvironment status and evaluated the efficacy of immunotherapy and chemotherapy in STS. The novel nomogram had good efficacy in predicting the prognosis of STS patients. Finally, a pan-cancer investigation verified the association of INFscore with prognosis and immunity.

**Conclusions:**

According to the present study, the risk stratification model can be used to evaluate STS prognosis, tumor microenvironment status, immunotherapy, and chemotherapy efficacy. The novel nomogram has an excellent predictive value. Thus, the INFscore and risk stratification model has potential value in assessing the prognosis and immune status of multiple malignancies.

## Introduction

Soft tissue sarcomas (STSs) are a heterogeneous group of tumors originating from mesenchymal tissue. STS accounts for approximately 0.8% of all adult malignancies, and there are over 70 histologic subtypes with the majority occurring in the trunk, extremities, and retroperitoneum (1). According to American Cancer Society data, 13,190 new STS cases and 5,130 fatalities will occur in the United States in 2022 (2). Although the incidence of STS is low, approximately 25-40% of STS patients will develop local recurrence or distant metastases even after radical resection (1). The 5-year survival rate for advanced STS is less than 20%, and effective therapies are limited (3, 4). The U.S. Food and Drug Administration (FDA) has approved inhibitors, such as regorafenib and pazopanib, for the clinical treatment of STS patients. However, the outcomes, such as objective remission rate (ORR), disease control rate (DCR), progression-free survival (PFS), and overall survival (OS), of inhibitors applied to the treatment of patients with advanced disease are not satisfactory (5, 6).

The immune system plays a crucial role in anticancer activity. However, immune escape has been identified in various malignancies (7). Immune checkpoint inhibitors (ICIs) are a promising therapy option for advanced malignancies (8, 9). Recently, many clinical trials have explored the efficacy and biomarkers of ICIs in treating STS. The SARC028 and Alliance A091401 clinical trials have suggested that cancer immunotherapy may improve the prognosis of STS patients (10, 11). ICIs for STS have been tested in approximately 20 clinical trials (12). Additionally, research on molecular indicators to forecast the clinical effectiveness of ICIs for STSs is underway. The following factors are expected to be biomarkers for predicting the efficacy of ICI treatment: cytokines in the tumor microenvironment; tumor infiltrating lymphocytes (TILs) and associated macrophages; and immune checkpoint proteins, such as programmed cell death-1 (PD-1), programmed cell death ligand-1 (PD-L1), cytotoxic T-lymphocyte associated protein 4 (CTLA-4), and major histocompatibility complex (MHC) (12, 13). However, the intricate and specific biological background of STS contributes to the sluggish and inadequate development of novel treatments (10, 14). As a result, it is important identify and develop novel biomarkers to predict and assess the prognosis of STS patients.

Evidence has demonstrated that inflammatory response-related genes can predict tumor prognosis and metastatic potentials in lung cancer and hepatocellular carcinoma (15, 16). However, the relationship between inflammatory response-related genes and STS has not been established. Based on the inflammatory response-related genes, we constructed a scoring system named inflammatory response-related gene score (INFscore) to evaluate the prognosis and immune status of STS. We developed and validated a novel nomogram and risk stratification model that includes the INFscore to evaluate STS patients. Furthermore, we demonstrated that the INFscore can be used to predict the efficacy of chemotherapy and immunotherapy in pan-cancer analysis.

## Patients and methods

### Patients and datasets for processing

RNA-seq data and clinical data for STS patients were downloaded from The Cancer Genome Atlas (TCGA) database (https://www.cancer.gov/). TCGA gene expression data were transformed into transcripts per kilobase million (TPM) format. Gene expression data for muscle and fat tissue samples (n=911) in Genotype-Tissue Expression (GTEx) Portal were downloaded from the University of California Santa Cruz (UCSC) Xena (https://xenabrowser.net/) for use as matched controls. The UCSC Xena browser is a publicly available browser for analysis and visualization of public datasets. Inflammatory response-related genes were obtained from the Gene Set Enrichment Analysis (GSEA) gene set (http://www.gsea-msigdb.org/gsea/index.jsp) (17).

### Patients and datasets for validation

We obtained gene expression profiles and clinical data from the GSE63155 independent cohort from the Gene Expression Omnibus (GEO) database (https://www.ncbi.nlm.nih.gov/geo/). We collected tissue samples from six STS patients admitted to the Tianjin Medical University’s Cancer Institute and Hospital between 2016 and 2019 to perform whole-exome sequencing (WES-seq) to determine their immune status. The retrospective investigation was performed in compliance with the Helsinki Declaration and was authorized by the Tianjin Medical University Cancer Institute and Hospital’s Ethics Committee (Approval No. E2019144). All patients provided a written informed consent. Tissue samples from six patients were genetically sequenced using WES-seq (the trial registration was NCT04126993) (18) by the Yuce Biotechnology Company. The exome sequencing process is described in detail in **Supplementary Data 1**.

### Acquisition of intersection genes and enrichment analysis

The limma package in R was used to identify differentially expressed genes (DEGs) between STS and normal tissue [with a false discovery rate (FDR) filter of 0.05 and a log fold change (FC) filter of 1]. Univariate Cox regression analysis was used to identify prognostic genes. The intersection genes between prognostic genes and DEGs were selected. Gene Ontology (GO) and Kyoto Encyclopedia of Genes and Genomes (KEGG) enrichment analyses were used to explore the related signaling pathways.

### Construction and validation of a prognostic inflammatory response-related gene signature and risk stratification model

We divided all STS patients into a training and validation set at a 1:1 ratio using the caret package in R. We performed least absolute shrinkage and selection operator (LASSO)-Cox in the training set to identify hub genes (19). We calculated the INFscore for each STS patient based on the hub genes using the following formula:


$$\text{INFscore}=\sum_{1}^{n}coefficient({\text{Gene}}_{\text{i}})\times expression({\text{Gene}}_{\text{i}})$$

We used receiver operating characteristic (ROC) curves and the area under the curve (AUC) value to evaluate the sensitivity and specificity of the INFscore in the training and validation sets. We divided the STS patients into high- and low-risk groups based on the median INFscore value. The overall survival was compared between the two groups using the log-rank test, and we validated the efficacy of the INFscore in the GSE16355 dataset.

### Construction and validation of the nomogram

Along with age, sex, tumor site, cancer type, margin status, and metastasis status, we included the INFscore as a variable in the univariate and multivariate Cox regression analyses. Based on the results of the independent prognostic factors from Cox regression analysis, we built a prognostic nomogram model. We generated ROC curves, estimated AUC values, and drew calibration and decision curve analysis (DCA) to validate the performance of the prognostic nomogram using the training and validation sets.

### Functional enrichment analysis

We performed Gene Set Variation Analysis (GSVA) (20) and GSEA to further elucidate the biological process differences between the high- and low-risk groups. The GSVA program in R (with logFC filter > 0.1, p-value 0.05) was used to compare the different biological processes between the two groups. GSEA is used to evaluate the distribution trend of genes in a predefined gene set in a gene table ranked by their relevance to phenotype, thereby assessing their contribution to the phenotype (21). The association between hub gene expression and the KEGG enrichment analysis in high- and low-risk groups was investigated using GSEA software (version 4.1.0). For the operation, we used the genome “c2.cp.kegg.v7.2.symbols.gmt.”, and nominal p-values of 0.05 and FDR q-values of 25% were considered significant.

### Immune microenvironment assessment

Because the INF-related genes were mainly enriched in the immune signaling pathway, we focused on the relationship between the INFscore and tumor immune microenvironment (TIME) in STS patients. Firstly, we used the GSEABase and GSVA packages in R to perform single-sample GSEA (ssGSEA) for 16 immune cell infiltration scores and 13 immune-related pathway scores in the high- and low-risk groups. The association between INFscore and tumor-infiltrating immune cells was examined by XCELL, TIMER, QUANTISEQ, MCPcounter, EPIC, CIBERSORT, and CIBERSORT-ABS (22). We then calculated and compared the stromal scores, immune scores, and Estimation of Stromal and Immune Cells in Malignant Tumor Tissues Using Expression Data (ESTIMATE) scores the between high- and low-risk groups. The association between risk assessment models and immune checkpoint-associated biomarkers was also investigated. Six patients from Tianjin Medical University Cancer Institute and Hospital were utilized for external validation to further investigate and validate immune cell infiltration in different risk strata in the real world.

### INFscore and chemotherapy sensitivity

The pRRophetic package in R selected 138 kinds of drugs from more than 700 cell lines in the Genomics of Drug Sensitivity in Cancer database (GDSC, https://www.cancerrxgene.org/) and developed a ridge regression algorithm to predict treatment responses. Semi-inhibitory concentrations (IC_50_) were calculated using the pRRophetic package in R to assess the sensitivity between high- and low-risk groups to common chemotherapy agents (we defined significance at p< 0.001).

### Utility of INFscore in pan-cancer analysis

Data from the pan-cancer cohort, including RNA-seq data, overall survival time, and survival status, were retrieved from TCGA database. A total of 10,792 samples from 32 types of malignancies were retrieved from TCGA database, and the INFscore was calculated. We assessed the variability of the INFscore in tumor tissue versus paraneoplastic tissue (or normal tissue), and we then assessed the relationship of the INFscore with the immune, stromal, and ESTIMATE scores. Finally, we explored the relationship between the INFscore and pan-cancer immune cell infiltration.

### Statistical analysis

For the statistical analyses, t tests were used to evaluate differences in quantitative data and regularly distributed variables, while Wilcoxon rank-sum tests were used to analyze differences in non-normally distributed variables. For correlation analysis, Spearman’s analysis was employed. The survival differences in the groups were compared using a log-rank test and illustrated by a Kaplan-Meier (K-M) survival plot. All statistical p-values were two-sided, and p< 0.05 indicated statistical significance. All data were processed using R software (version 4.1.1).

## Results

**Figure 1:**
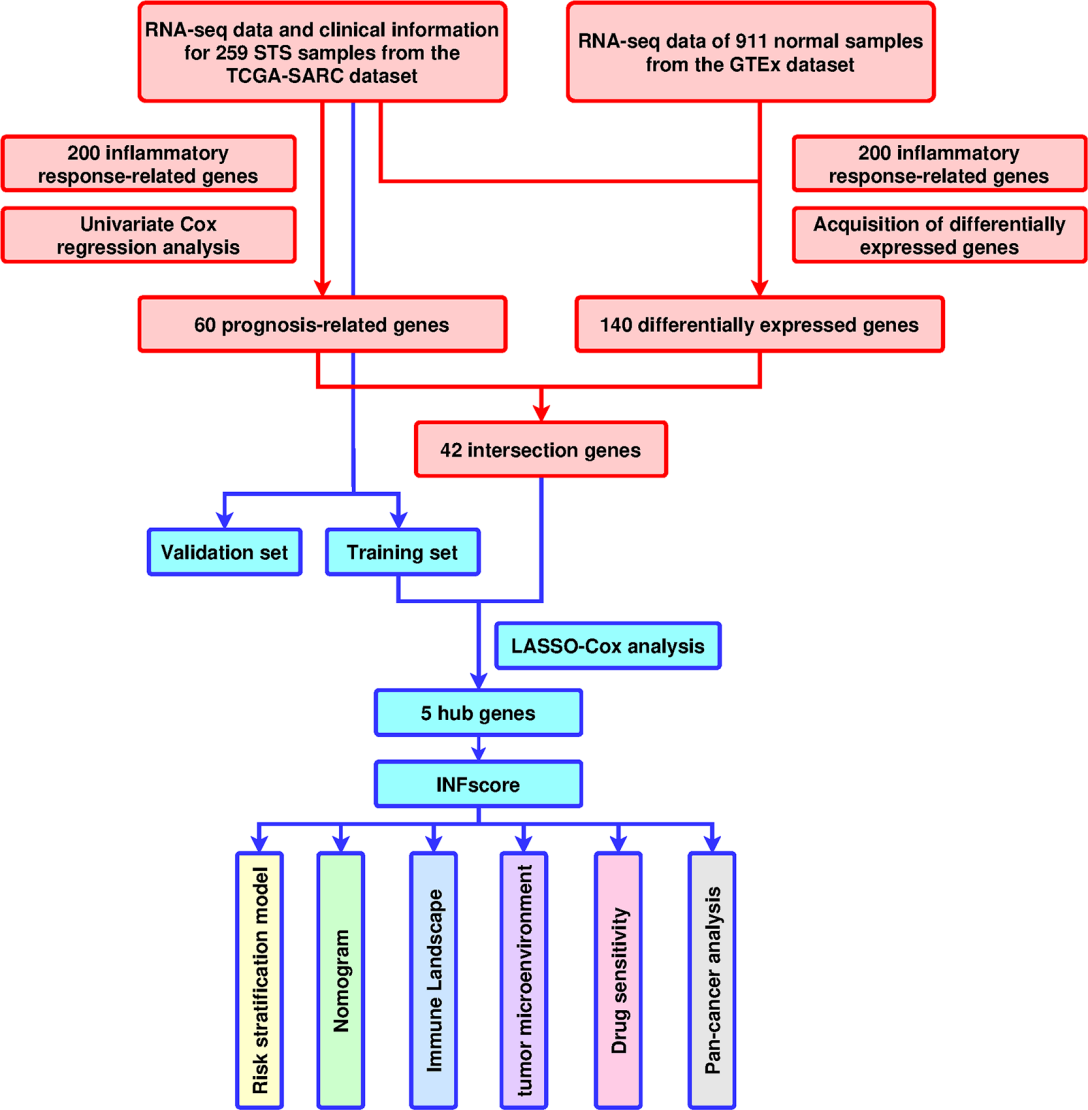
Design and workflow of the study. TCGA, The Cancer Genome Atlas; GTEX; GO, Gene Ontology; KEGG, Kyoto Encyclopedia of Genes and Genomes; LASSO, least absolute shrinkage and selection operator; STS, soft tissue sarcomas; GSVA, Gene Set Variation Analysis; ssGSEA, single-sample GSEA; ROC, receiver operating characteristic; INFscore, inflammatory response-related gene score.

In total, 259 STS patients from TCGA-SARC dataset were included, and the clinicopathological characteristics are listed in **Table 1**. A total of 911 RNA-seq data of muscle and adipose tissue were downloaded from the GTEx database. Moreover, 200 inflammatory response-related genes were downloaded from the GSEA database and are listed in **Supplementary Table S1**. The GSE63157 dataset, including 46 sarcoma sample files, was used as the validation dataset. Data from TCGA, GTEx, GSEA, and GEO are all publicly available, and the datasets were used following the data access policies and publishing standards. **Figure 1** depicts the flow chart for this study.

**Table 1:** Clinicopathological features of 259 soft tissue sarcoma patients.

	Total set (n = 259)	Training set (n = 130)	Validation set (n = 129)	χ^2^	*P*
Age, years				0.664	0.415
≤65	159	83	76		
>65	100	47	53		
Sex				0.309	0.578
Male	118	57	61		
Female	141	73	68		
Histological type				6.857	0.144
DLS	58	30	28		
LMS	104	49	25		
MFS	25	15	10		
OTHER	51	30	21		
UPS	21	6	15		
Tumor site				1.994	0.158
Extremities	85	48	37		
Other	174	82	92		
Margin status				0.205	0.650
Negative or Unknown	186	95	91		
Positive	73	35	38		
Metastasis				1.361	0.243
M0	191	100	91		
M1	68	30	38		

LMS, leiomyosarcoma; UPS, undifferentiated pleomorphic sarcoma; LPS, Liposarcoma; MFS, Myxofibrosarcoma.

### Identification of inflammatory response-related hub genes in STS patients

**Figure 2:**
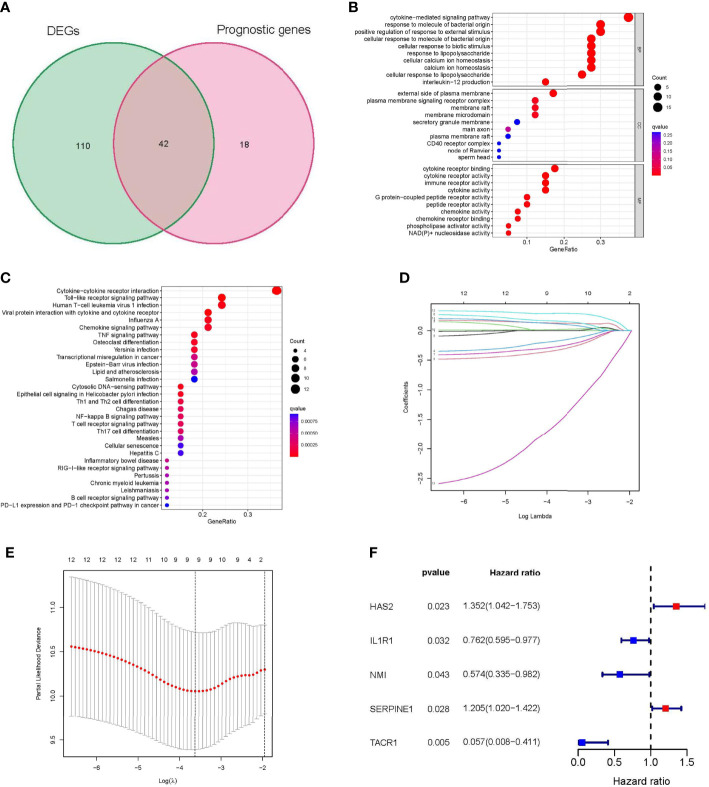
Identification of Hub genes in STS. **(A)** Venn diagram showing 42 prognostic differentially expressed genes (DEGs). **(B)** Functional annotation of intersection genes using Gene Ontology (GO) enrichment analysis. **(C)** Functional annotation of intersection genes using Kyoto Encyclopedia of Genes and Genomes (KEGG) enrichment analysis. **(D, E)** The results of LASSO analysis. **(F)** univariate Cox regression analysis of 5 hub genes. DEG: differentially expressed gene; GO: Gene Ontology; KEGG: Kyoto Encyclopedia of Genes and Genomes; LASSO: least absolute shrinkage and selection operator; STS: soft tissue sarcomas.

A total of 152 differentially expressed inflammatory response-related genes were found (**Supplementary Figures S1A, B**), and univariate Cox regression analysis indicated that 60 inflammatory response-related genes were associated with the prognosis of STS patients (**Supplementary Figure S1C**). Among them, 42 prognostic DEGs were identified by the Venn diagram (**Figure 2A**). GO and KEGG enrichment analyses showed that immune-related biological functions and signaling pathways played an important role in the inflammatory response process (**Figures 2B, C**). In addition, 259 sarcoma patients were randomly separated into training and validation sets at a 1:1 ratio. The expression profiles of the above 42 genes were analyzed using LASSO-Cox regression analysis in the training set. The following five hub genes were identified: hyaluronan synthase 2 (HAS2), interleukin 1 receptor type 1 (IL1R1), N-Myc and STAT interactor (NMI), serpin family E member 1 (SERPINE1), and tachykinin receptor 1 (TACR1) (**Figures 2D–F**).

### Establishment of prognostic models by the INFscore

**Figure 3:**
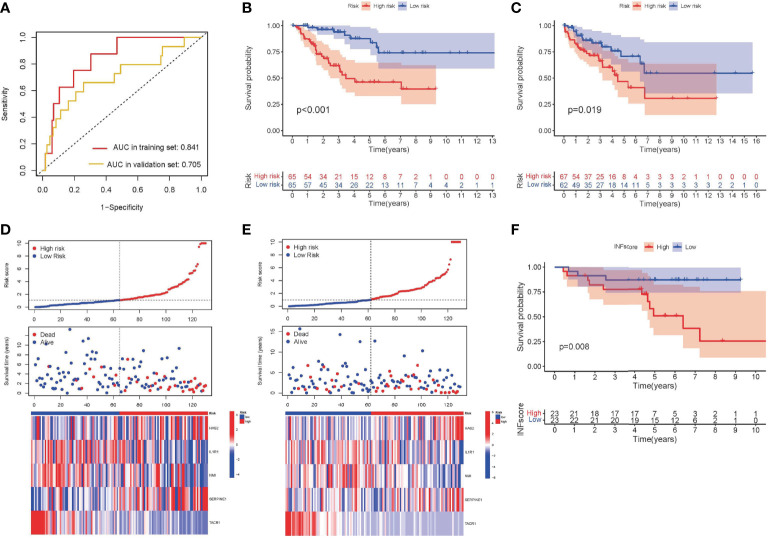
Development and validation of INFscore and risk stratification model. **(A)** ROC analysis of INFscore in predicting prognosis. The analysis of the training set is marked in red and that of the validation set is marked by yellow. **(B)** Kaplan–Meier analysis of patients in the high risk and low risk groups in the training set. **(C)** Kaplan–Meier analysis of patients in the high risk and low risk groups in the validation set. **(D)** Risk score plot, survival status plot, and expression pattern of 5 hub genes between high and low-risk groups in the training set. **(E)** Risk score plot, survival status plot, and expression pattern of 5 hub genes between high and low-risk groups in the validation set. **(F)** Kaplan–Meier analysis of patients in the high risk and low risk groups in validation set GSE63155. ROC: receiver operating characteristic; INFscore: inflammatory response-related gene score.

The INFscore was calculated as follows: INFscore = 0.226 × Expression(HAS2) + (-0.459) × Expression(IL1R1) + (-0.547) × Expression(NMI) + 0.188 × Expression(SERPINE1) + (-2.868) × Expression(TACR1). The AUC values of the training and validation sets were 0.841 and 0.705, respectively (**Figure 3A**). According to the median cutoff point in the training set (Median INFscore = 1.090), 259 STS patients were used to establish a risk stratification model (high- and low-risk groups). Survival analysis was performed using log-rank tests and by plotting K-M survival curves (**Figures 3B, C**). The risk score plots and survival status plots of the training and validation sets illustrated that overall survival decreased with increasing INFscore (**Figures 3D, E**). We then validated the risk stratification model using the GSE63155 dataset as an external database (**Figure 3F**).

### Subgroup analyses

A survival analysis of different subgroups of STS patients based on their clinicopathological characteristics was performed to further validate the prognostic significance of high- and low-risk groups. For STS patients with different clinicopathological features, patients in the high-risk category had a worse outcome (**Supplementary Figure S2**).

### Establishment and verification of INF-related nomogram

**Figure 4:**
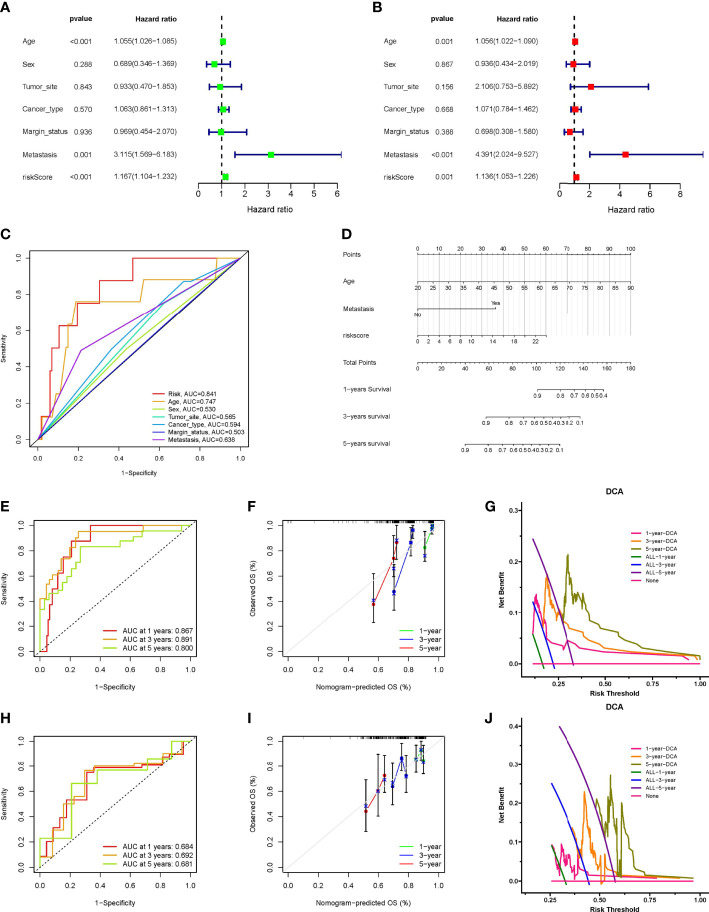
Construction and verification of Nomogram. **(A)** The results of univariate Cox regression analysis. **(B)** The results of multivariate Cox regression analysis. **(C)** Comparison of INFscore and other clinicopathological features predicting prognosis by ROC analysis. **(D)** The nomogram included age, INFscore, and metastasis status for predicting the 1-, 3-, and 5-year survival rates of STS patients. **(E)** ROC curves of nomogram for predicting 1-, 3-, and 5-year prognosis in the training set. **(F)** Calibration curves of the nomogram 1-, 3-, and 5-year prognosis in the training set. **(G)** DCA of the nomogram 1-, 3-, and 5-year prognosis in the training set. **(H)** ROC curves of nomogram for predicting 1-, 3-, and 5-year prognosis in the validation set. **(I)** Calibration curves of the nomogram 1-, 3-, and 5-year prognosis in the validation set. **(J)** DCA of the nomogram 1-, 3-, and 5-year prognosis in the validation set. DCA: Decision curve analysis; ROC: receiver operating characteristic; STS: soft tissue sarcomas.

Cox regression analysis indicated that the INFscore was an independent predictive factor for STS patients in the training set (**Figures 4A, B**), and the AUC value of the INFscore was higher than those other clinicopathological features (**Figure 4C**). Other independent prognostic factors in STS patients included age and metastatic status (M1 vs. M0). We next established the prognostic nomogram for STS patients (**Figure 4D**). As an example, the total score for a 48year-old STS patient with distant metastases and an INFscore of 10 was calculated by summing the scores for each variable, resulting in a total score of 100 with 1-year, 3-year, and 5-year survival rates of 0.90, 0.59, and 0.35, respectively. The ROC, calibration, and DCA curves showed that the nomogram had good predictive ability in the training set (**Figures 4E–G**). The ROC curves, calibration curves, and DCA in the validation set also confirmed the predictive ability of the nomogram (**Figures 4H–J**).

### TME landscape between high- and low-risk STS patients

**Figure 5:**
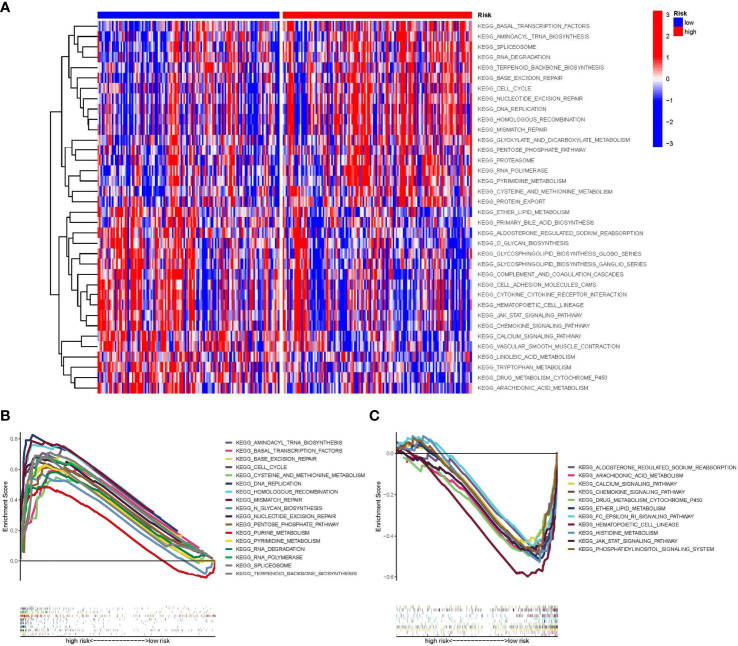
Gene set variation analysis (GSVA) and Gene set enrichment analysis (GSEA). **(A)** The results of the heat map are presented by GSVA scores, demonstrating the characteristic signaling pathways that differ in high and low-risk groups. **(B)** GSEA plot showing signaling pathways activated in the high-risk group. **(C)** GSEA plot showing signaling pathways activated in the low-risk group. GSVA, Gene set variation analysis; GSEA, Gene set enrichment analysis.

We utilized GSVA to investigate the biological function differences between high- and low-risk groups (**Figure 5A**). The high-risk group was mainly enriched in cell cycle regulation (e.g., cell cycle and DNA replication), and the low-risk group was related primarily to substance metabolism (e.g., arachidonic acid metabolism) and the chemokine signaling pathway. We used GSEA to further evaluate the differences in biological status between the high- and low-risk groups as well as to separate the types of expression patterns of specific sets of important genes (**Figures 5B, C**). Cell cycle regulation-related signaling pathways (e.g., DNA replication and cell cycle) were highly expressed in STS patients in the high-risk group, whereas substance metabolism (e.g., arachidonic acid metabolism and cytochrome p450 drug metabolism) and signaling pathway transduction [e.g., Janus kinase-signal transducer and activator of transcription (JAK-STAT) signaling pathway] were highly expressed in STS patients in the low-risk group.

### Immune landscape between high- and low-risk STS patients

**Figure 6:**
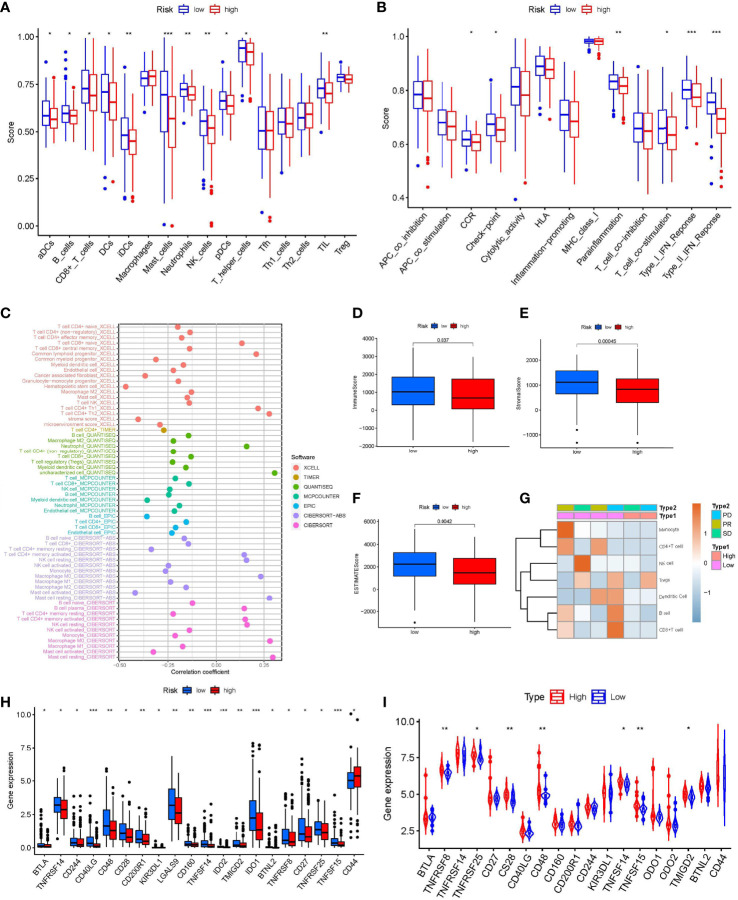
Immune Landscape Characteristics. **(A)** The box plot of immunologic cell analysis between high and low-risk group. **(B)** The box plot of immunologic function analysis between high and low-risk group. **(C)** The results of Spearman correlation analysis showed a negative correlation between INFscore and immune cell infiltration in STS patients. **(D–F)** Immune score, Stromal score, and ESTIMATE score in high and low-risk group. **(G)** Immune cells infiltration heat map of the six patients from Tianjin Medical University Cancer Institute and Hospital. **(H)** Comparison of immune checkpoint blockade–related genes expression levels in STS patients with the high and low-risk group. **(I)** The expression levels of immune checkpoint associated genes in the high-risk and low-risk groups in the validation set gse63155 followed the same pattern. Asterisks represent the statistical *P*‐values (*P< 0.05; **P< 0.01; ***P< 0.001). STS: soft tissue sarcomas.

Because the INF-related genes were mainly enriched in the immune signaling pathway, we focused on the relationship between the INFscore and TIME in STS patients. To assess the abundance of immune cell infiltration between the high- and low-risk groups, we analyzed 16 immune cells and 13 immune function scores using ssGSEA (**Figures 6A, B**). The results of ssGSEA showed that dendritic cells (DCs), activated DCs (aDCs), B cells, CD8^+^ T cells, immature DCs (iDCs), mast cells, neutrophils, natural killer (NK) cells, plasmacytoid DCs (pDCs), T helper cells, and TILs were higher in the low-risk group than in the high-risk group (p < 0.05). The results of ssGSEA also indicated that cytokine-cytokine receptor (CCR), checkpoint, parainflammation, T cell costimulation, and type I/II IFN response were higher in the low-risk group than in the high-risk group (p < 0.05). At the same time, we applied seven other methods to evaluate the relationship between immune cell infiltration and the INFscore (**Figure 6C**; **Supplementary Figure S3**). According to the results of the seven algorithms, the INFscore had a negative association with the degree of infiltration of most immune cells. From an overall perspective, the Kruskal-Wallis test was used to assess the differences in immune scores, stromal scores, and ESTIMATE scores between the high- and low-risk STS patient groups. Compared to the high-risk group, the immune score (p = 0.037), stromal score (p< 0.001), and ESTIMATE score (p< 0.0001) were significantly higher in the low-risk group than the high-risk group (**Figures 6D–F**). We separated six patients from the Tianjin Medical University Cancer Institute and Hospital into two groups based on their INF scores, namely, low-risk (four samples) and high-risk (two samples), and we generated an immune cell infiltration heatmap using these samples (**Figure 6G**). The real world data verified that the low-risk group had more immune cell infiltration than the high-risk group.

### The role of the INFscore in predicting immunotherapeutic benefits

Because the checkpoint immune function scores were higher in the low-risk group than in the high-risk group, we analyzed the variability of 47 known immune checkpoint-associated genes (**Supplementary Table S2**) in the high- and low-risk groups (**Figure 6H**). The expression of B and T lymphocyte associated (BTLA), TNF receptor superfamily member 8 (TNFRSF8), TNFRSF14, TNFRSF25, CD27, CD28, CD40 ligand (CD40LG), CD48, CD160, CD200 receptor 1 (CD200R1), CD244, killer cell immunoglobulin like receptor (KIR3DL1), galectin 9 (LGALS9), TNF superfamily member 14 (TNFSF14), TNFSF15, indoleamine 2,3-dioxygenase 1 (IDO1), IDO2, transmembrane and immunoglobulin domain containing 2 (TMIGD2), and butyrophilin like 2 (BTNL2) in the low-risk group was higher than that in the high-risk group. The expression levels of immune checkpoint-associated genes in the high- and low-risk groups in the GSE63155 validation set followed a similar pattern (**Figure 6I**).

### Risk classification and chemotherapy sensitivity

**Figure 7:**
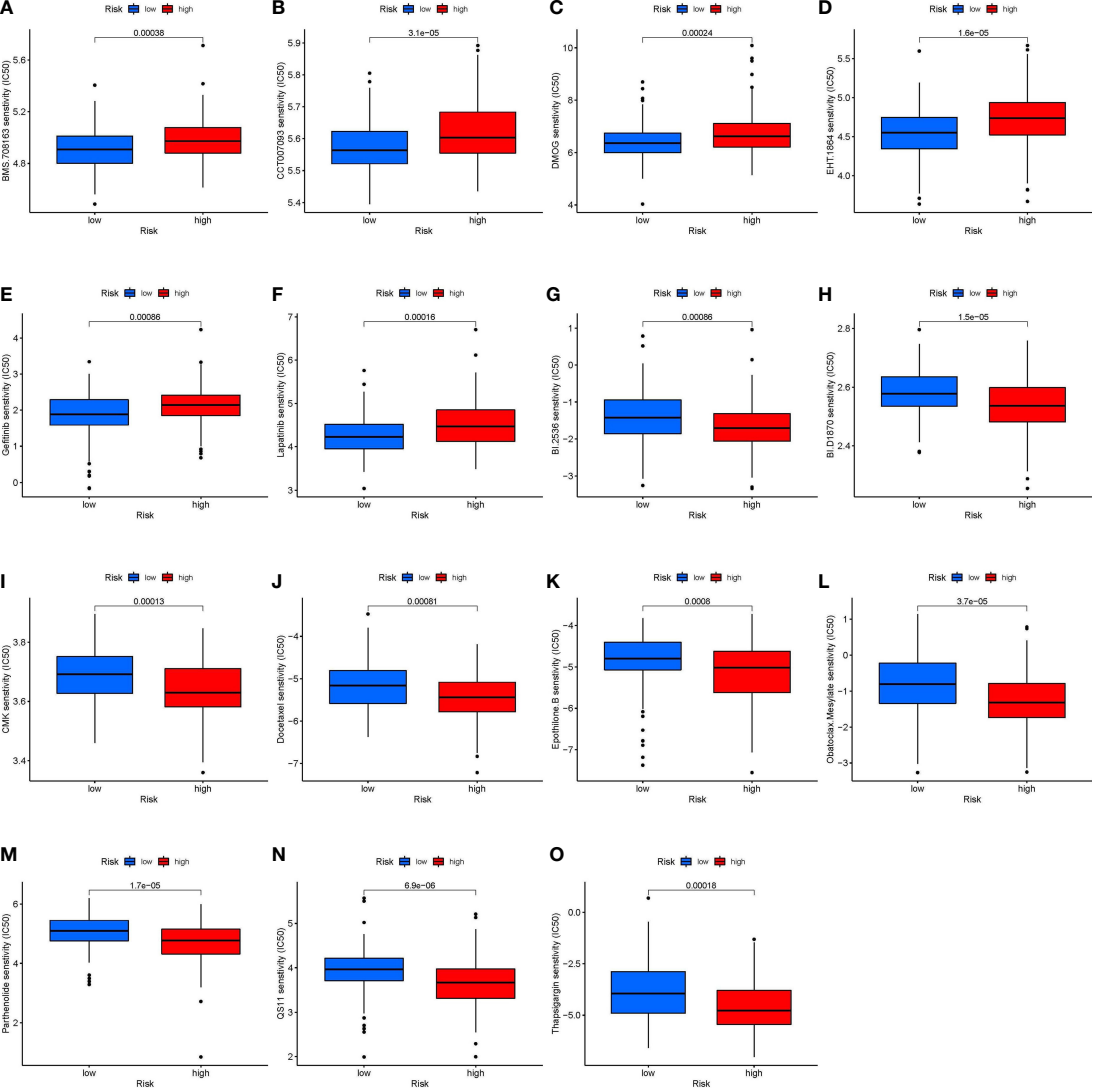
Correlation between risk stratification models and chemotherapy sensitivity in STS. The IC50 of **(A)** BMS.708163; **(B)** CCT007093; **(C)** DMOG **(D)** EHT.1864; **(E)** Gefitinib; **(F)** Lapatinib; **(G)** BI.2536; **(H)** BI.D1870; **(I)** CMK **(J)** Docetaxel; **(K)** Epothilone. B; **(L)** Obatoclax. Mesylate; **(M)** Parthenolide; **(N)** QS11; and **(O)** Thapsigargin in the high-risk and low-risk groups. BMS.708163: γ secretase inhibitor; CCT007093: Wild type p53 inducible phosphatase (WIP1) inhibitor; DMOG: Dimethyloxalylglycine; EHT.1864: RAC inhibitor; BI.2536: Polo Like Kinase 1 (PLK1) inhibitors; BI.D1870: pan-RSK inhibitor; CMK: RSK2 inhibitor; QS11: ADP Ribosylation Factor GTPase Activating Protein 1 (ARFGAP1) inhibitor.

The Wilcox test revealed significant differences (p < 0.001) between the high and low-risk groups for 15 chemotherapeutic medications when the IC_50_ levels of 138 medicines were measured in STS patients (**Figure 7**). The data showed that the IC_50_ levels of the following medicines were significantly higher in the high-risk group than in the low-risk group: BMS.708163 (γ secretase inhibitor, **Figure 7A**), CCT007093 [wild-type p53 inducible phosphatase (WIP1) inhibitor, **Figure 7B**], DMOG (dimethyloxalylglycine, **Figure 7C**), EHT.1864 (RAC inhibitor, **Figure 7D**), gefitinib (**Figure 7E**), and lapatinib (**Figure 7F**). In contrast, the IC_50_ levels of the following medicines were significantly higher in the low-risk group than in the high-risk group: BI.2536 [polo like kinase 1 (PLK1) inhibitors, **Figure 7G**], BI-D1870 (pan-RSK inhibitor, **Figure 7H**), CMK (RSK2 inhibitor, **Figure 7I**), docetaxel (**Figure 7J**), epothilone B (**Figure 7K**), obatoclax mesylate (**Figure 7L**), parthenolide (**Figure 7M**), QS11 [ADP ribosylation factor GTPase activating protein 1 (ARFGAP1) inhibitor, **Figure 7N**], and thapsigargin (**Figure 7O**).

### Utility of the INFscore in pan-cancer analysis

**Figure 8:**
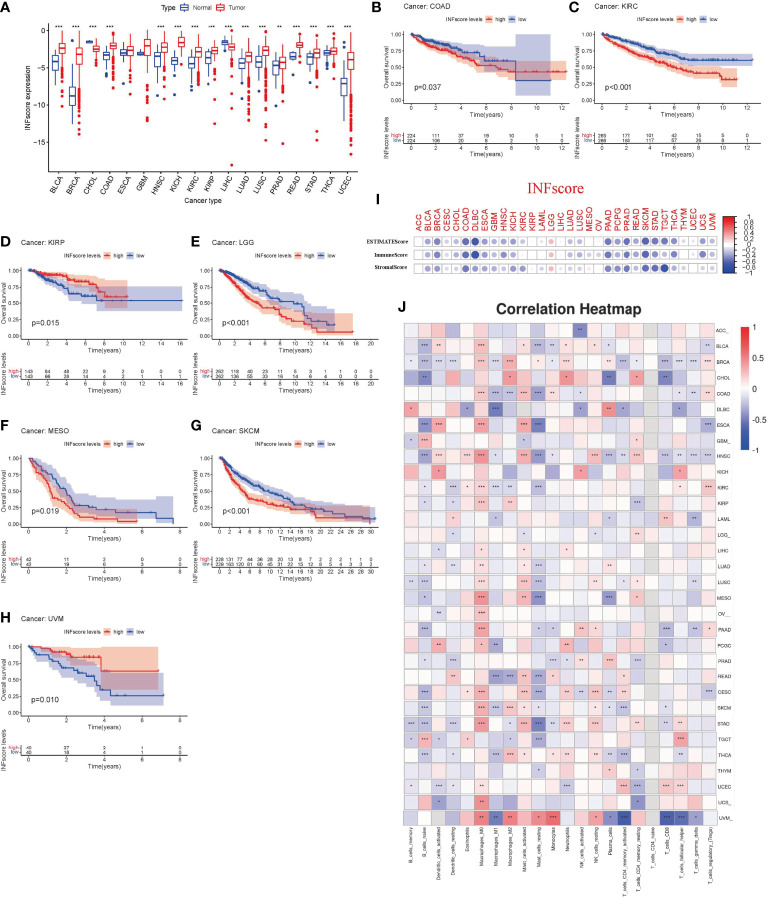
The utility of INFscore and risk stratification models in pan-cancer. **(A)** Differential INFscore between tumor and normal tissues in The Cancer Genome Atlas (TCGA) database. Red color represents cancer samples and blue color represents normal samples. **(B–H)** Association of INFscore with patient overall survival in pan-cancer. High INFscore predicts poor overall survival of **(B)** Colon adenocarcinoma (COAD), **(C)** Kidney renal clear cell carcinoma (KIRC), **(D)** Kidney renal papillary cell carcinoma (KIRP), **(E)** Brain Lower Grade Glioma (LGG), **(F)** Mesothelioma (MESO), **(G)** Skin Cutaneous Melanoma (SKCM), and **(H)** Uveal Melanoma (UVM). **(I)** Immune score, Stromal score, and ESTIMATE score in 32 different types of tumors. **(J)** the correlation between the degree of 22 immune cell infiltration and INFscore in 32 types of cancer. Asterisks represent the statistical *P*‐values (*P< 0.05; **P< 0.01; ***P< 0.001). TCGA, The Cancer Genome Atlas; INFscore, inflammatory response-related gene score; ACC, Adrenocortical carcinoma; BLCA, Bladder Urothelial Carcinoma; BRCA, Breast invasive carcinoma; CHOL, Cholangiocarcinoma; COAD, Colon adenocarcinoma; DLBC, Lymphoid Neoplasm Diffuse Large B-cell Lymphoma; ESCA, Esophageal carcinoma; GBM, Glioblastoma multiforme; HNSC, Head and Neck squamous cell carcinoma; KICH, Kidney Chromophobe; KIRC, Kidney renal clear cell carcinoma; KIRP, Kidney renal papillary cell carcinoma; LAML, Acute Myeloid Leukemia; LGG, Brain Lower Grade Glioma; LIHC, Liver hepatocellular carcinoma; LUAD, Lung adenocarcinoma; LUSC, Lung squamous cell carcinoma; MESO, Mesothelioma; OV, Ovarian serous cystadenocarcinoma; PAAD, Pancreatic adenocarcinoma; PCGC, Pheochromocytoma and Paraganglioma; PRAD, Prostate adenocarcinoma; READ, Rectum adenocarcinoma; CESC, Cervical squamous cell carcinoma and endocervical adenocarcinoma; SKCM, Skin Cutaneous Melanoma; STAD, Stomach adenocarcinoma; TGCT, Testicular Germ Cell Tumors; THCA, Thyroid carcinoma; THYM, Thymoma; UCEC, Uterine Corpus Endometrial Carcinoma; UCS, Uterine Carcinosarcoma; UVM, Uveal Melanoma.

To further determine the role of the INFscore in various cancer types, we calculated the INFscore for 10,792 samples from 32 cancer types. We found higher INFscores in bladder urothelial carcinoma (BLCA), breast invasive carcinoma (BRCA), cholangiocarcinoma (CHOL), colon adenocarcinoma (COAD), head and neck squamous cell carcinoma (HNSC), kidney chromophobe (KICH), kidney renal clear cell carcinoma (KIRC), kidney renal papillary cell carcinoma (KIRP), liver hepatocellular carcinoma (LIHC), lung adenocarcinoma (LUAD), prostate adenocarcinoma (PRAD), rectum adenocarcinoma (READ), stomach adenocarcinoma (STAD), thyroid carcinoma (THCA), and uterine corpus endometrial carcinoma (UCEC) than in paraneoplastic or normal tissues by the Wilcox test (**Figure 8A**). K-M survival analysis showed that in COAD (p = 0.037), KIRC (p < 0.001), KIRP (p = 0.015), brain lower-grade glioma (LGG, p < 0.001), mesothelioma (MESO, p = 0.019), skin cutaneous melanoma (SKCM, p < 0.001), and uveal melanoma (UVM, p = 0.010), the high INFscore group had a worse prognosis than the low INFscore group (**Figures 8B–H**). To further assess the relationship between the INFscore and immunity in pan-cancer, we calculated the immune score, stromal score, and ESTIMATE score, which indicated that the INFscore was negatively correlated with the immune score, stromal score, and ESTIMATE score in 32 different types of tumors (**Figure 8I**). In addition, we further analyzed the correlation between the degree of infiltration of 22 immune cells and the INFscore in 32 types of cancer (**Figure 8J**).

## Discussion

Several obstacles exist in the treatment of STS, including the heterogeneity of STS (1), the presence of micrometastases in some STS patients before resection (23), unresectable lesions (24), and chemotherapy resistance (25). Therefore, novel molecular indicators are required to forecast STS patients’ prognosis and create tailored treatment regimens that guide efficient antitumor responses. Some inflammatory response markers have previously been used to predict prognosis in STS patients (26, 27). The prognosis of retroperitoneal sarcoma can be determined by serum markers of the innate inflammatory response [e.g., neutrophil-to-lymphocyte ratio (NLR) and C-reactive protein (CRP)] (26). Moreover, Kobayashi et al. confirmed that NLR values can be used to predict the response to pazopanib and the prognosis in STS patients (27). However, studies on the inflammatory response-related gene markers as prognostic biomarkers in STS patients have not been reported. In the present study, we found that inflammatory response hub genes regulated metabolism, cell cycle, and the immune microenvironment in STS.

Firstly, we identified five hub genes (HAS2, IL1R1, NMI, SERPINE1, and TACR1) and calculated the INFscore for each patient, and multivariate Cox regression analysis indicated that the INFscore was an independent prognostic factor. Finally, to better generalize the INFscore to the clinic, we created an inflammatory response-related nomogram based on the independent prognostic factors in the training set for the first time. Thus, using the nomogram, clinicians can provide potential guidance and value for clinical work through stratum-by-stratum analysis and validation.

In oncology and immunology, the association among inflammation, immunity, and tumorigenesis has been investigated in recent years (28, 29). GO and KEGG analyses showed that inflammatory response-related prognostic DEGs were closely related to immunity in STS patients. To further explore the relationship between them, we analyzed the immune landscape in STS patients with different risk stratification. Immunogenic tumors are known as “hot tumors,” and non-immunogenic tumors are known as “cold tumors.” A “hot tumor” is defined as one in which the cancer cells are surrounded by a high number of immune cells that can recognize the cancer cells, while the contrary is valid for a “cold tumor” (30). The present study identified high and low INFscore risk groups with specific patterns associated with different anticancer immunity. The low-risk STS patients were characterized by immune activation and infiltrated immune cells, corresponding to a “hot tumor.” STS is considered a “cold tumor” with relative immunogenicity (31); however, there are still patients who benefit from immunotherapy, and the present study investigated such patients for potential benefits. Patients with STS in the high-risk group were characterized by immunosuppression, corresponding to a “cold tumor.” We utilized the six patients to corroborate the inherent variability and confirmed that the patients in the high-risk group were immune deserts while the patients in the low-risk group had “hot tumors”. Previous studies have confirmed that hot tumors can benefit from immunotherapy (30, 32). Thus, these results suggested that patients in the low-risk group may respond to immunotherapy based on the results of the immune landscape study. Surprisingly, the immune cell function score analysis revealed that the checkpoint score was higher in the low-risk group compared to the high-risk group. In addition, differential analysis of the 47 known immune checkpoint-associated genes in the risk groups indicated that 19 immune checkpoint-associated genes were highly expressed in the low-risk group, which verified that STS patients in the low-risk category were more sensitive to ICIs. These tendencies were consistent with the GSE61355 dataset.

To date, chemotherapy is a cornerstone in treating STS. However, chemotherapy-resistance and toxic side effects have become major obstacles for the use of chemotherapy (33, 34). Because STS has a high degree of heterogeneity (1), selecting appropriate and sensitive chemotherapeutic agents is especially important. The present study found that patients in the low-risk group were more sensitive to tyrosine kinase inhibitors but that patients in the high-risk group were more sensitive to paclitaxel-like drugs, which induce and promote the polymerization of microtubule proteins. Interestingly, chemotherapy sensitivity was associated with high expression of signaling pathways in different risk groups. Both GSVA and GSEA confirmed that cell cycle-related signaling pathways were more highly expressed in the high-risk group than the low-risk group. Therefore, we inferred that patients in the high-risk group were more sensitive to cell cycle-specific agents (CCSAs). GSEA and GSVA indicated that the JAK-STAT signaling pathway was highly expressed in the low-risk group. JAK-STAT consists of three components, namely, tyrosine kinase-associated receptors receiving the signal, tyrosine kinases delivering the signal, and transcription factors producing the effect (35). Previous studies have confirmed that differentiation of sarcoma disease characteristics and subtype classification facilitate the selection of appropriate targeted agents, thereby improving patient prognosis (36, 37). In the present study, gefitinib and lapatinib, which target tyrosine kinases, were the more sensitive chemotherapeutic agents in the low-risk group. Therefore, the risk stratification model in the present study is an excellent candidate to predict chemotherapy sensitivity. Finally, we elaborated the prognostic significance of the INFscore in other cancer types. The findings of the pan-cancer analysis further confirmed that the INFscore is closely associated with immunity in other cancers.

The present study had several limitations. Although the present findings were based on publicly available databases that have been validated using various internal and external datasets, external validation with a large sample is still required. The hub genes in the present study were based on TCGA-SCAR database, and the INFscore was calculated in pan-cancer, which only obtained the general direction and a rough assessment of the relationship between inflammation and immunity in pan-cancer. This method had certain shortcomings and requires further analysis and validation.

## Conclusion

In the present study, we comprehensively evaluated the prognosis of 259 STS patients based on validation of INF-related genes. We established a nomogram with good predictive power by internal validation. In addition, we analyzed the TIME landscape, immune landscape, and chemotherapy drug sensitivity of patients with different risks for STS by a risk stratification model, providing a reference value for selecting appropriate and more effective treatment modalities. Finally, we further explored the value of the INFscore in pan-cancer.

## Data availability statement

The original contributions presented in the study are included in the article/**Supplementary Material**. Further inquiries can be directed to the corresponding authors.

## Ethics statement

The studies involving human participants were reviewed and approved by Tianjin Cancer Hospital Medical Ethics Committee. The patients/participants provided their written informed consent to participate in this study.

## Author contributions

All authors contributed to the planning and design of the study ZL, JW, HZ, and JY were involved in review of the raw data and directly involved in the analysis. ZL and JW provided analytical feedback based on aggregated results. ZL and HZ drafted the manuscript, with input from all authors. ZL was responsible for the chart making. LL, ZF, HC, ZL, and HZ revised the manuscript. All authors provided substantive review and commentary on multiple drafts and approved the final version. JW and JY supervised the study.

## Funding

This study is funded by the Science & Technology Program of Chengde (No. 202109A041).

## Acknowledgments

We are very grateful for the contributions of TCGA, GTEx, GSEA, and GEO databases that provide information on cancer research, as well as all colleagues involved in the study.

## Conflict of interest

The authors declare that the research was conducted in the absence of any commercial or financial relationships that could be construed as a potential conflict of interest.

## Publisher’s note

All claims expressed in this article are solely those of the authors and do not necessarily represent those of their affiliated organizations, or those of the publisher, the editors and the reviewers. Any product that may be evaluated in this article, or claim that may be made by its manufacturer, is not guaranteed or endorsed by the publisher.

## Supplementary material

Supplementary Figure 1 Expression difference analysis and univariate Cox prognostic analysis. **(A)** Heat map of inflammatory response-related genes with significant differences. **(B)** Volcano plot of inflammatory response-related genes, green indicates downregulated genes, and red indicates upregulated genes. Black dots represent genes that are not differentially expressed in STS tissues and normal tissues, while green dots and red dots represent genes that are down- and up-regulated in cancer samples, respectively. **(C)** The forest plot shows 60 prognostic inflammatory response-related genes in terms of p-value, odds ratio (OR), and 95% confidence interval (CI). OR: odds ratio; CI: confidence interval.

Supplementary Figure 2 Prognostic value of risk stratification models in multiple subgroups of soft tissue sarcoma (STS) patients. **(A)** STS patient with age less than or equal to 65 years old, **(B)** STS patient with age more than 65 years old, **(C)** STS patient with histological type of DLS, **(D)** STS patient with histological type of LMS, **(E)** STS patient with histological type of MFS, **(F)** STS patient with other histological types, **(G)** STS patient with histological type of UPS, **(H)** STS patients with negative margin status, **(I)** STS patients with positive margin status, **(J)** STS patients without metastasis, **(K)** STS patients with distant metastasis, **(L)** STS patients with other Races, **(M)** STS patients with White, **(N)** STS patients with female, **(O)** STS patients with male, **(P)** STS patients with tumor sites in the extremities, **(Q)** STS patients with other tumor sites. LMS: leiomyosarcoma; UPS: undifferentiated pleomorphic sarcoma; LPS: Liposarcoma; MFS: Myxofibrosarcoma; STS: soft tissue sarcomas.

Supplementary Figure 3 The correlation between INFscore and immune cell infiltration. **(A1–A19)** Results of XCELL algorithm analysis of immune cell infiltration. **(B)** Results of TIMER algorithm analysis of immune cell infiltration, **(C1–C8)** Results of QUANTISEQ algorithm analysis of immune cell infiltration, **(D1–D7)** Results of MCPcounter algorithm analysis of immune cell infiltration, **(E1–E4)** Results of EPIC algorithm analysis of immune cell infiltration, **(F1–F12)** Results of CIBERSORT algorithm analysis of immune cell infiltration, **(G1–G11)** Results of CIBERSORT-ABS algorithm analysis of immune cell infiltration.
