# Supplementary material for: A novel inflammatory signature for evaluating immune microenvironment status in soft tissue sarcoma
Source: Front Oncol. 2022 Oct 13;12:990670. doi: 10.3389/fonc.2022.990670 (PMC9609423; doi:10.3389/fonc.2022.990670)
Supplement: Supplementary file 1 [file DataSheet_1.docx]

**Supplementary method**

Genomic DNA from formalinfixed,paraffin-embedded (FFPE) sections from biopsy samples or whole blood control samples were extracted using the Gene Read DNA FFPE Kit (Qiagen,Germantown, MD, USA) and the Mag-Bind Blood and Tissue DNA HDQ 96 Kit (Qiagen), respectively. Library preparations were performed using the KAPA Library Quantification Kit (Roche, Indianapolis, IN,USA), and target enrichment was performed using the Target Seq Enrichment Kit (iGene Tech, Beijing, China), and sequencing was performed on a NovaSeq (Illumina, San Diego, CA, USA). The raw reads of WES-seq were processed using SOAPnuke (version1.5.6, parameters: -l 20 -q 0.1 -n 0.1) to remove ambiguous reads and/or low-quality reads. These qualified sequence reads were then aligned to the human reference genome (UCSC hg38) using BWAmem (BWA, version 0.7.12).
