# Supplementary material for: A novel inflammatory signature for evaluating immune microenvironment status in soft tissue sarcoma
Source: Front Oncol. 2022 Oct 13;12:990670. doi: 10.3389/fonc.2022.990670 (PMC9609423; doi:10.3389/fonc.2022.990670)
Supplement: Supplementary file 2 [file Table_1.docx]

| Supplementary table 1. Inflammatory response-related genes | | | |
| --- | --- | --- | --- |
| ABCA1 | CYBB | IRF7 | PTGER2 |
| ABI1 | DCBLD2 | ITGA5 | PTGER4 |
| ACVR1B | EBI3 | ITGB3 | PTGIR |
| ACVR2A | EDN1 | ITGB8 | PTPRE |
| ADM | EIF2AK2 | KCNA3 | PVR |
| ADORA2B | EMP3 | KCNJ2 | RAF1 |
| ADRM1 | ADGRE1 | KCNMB2 | RASGRP1 |
| AHR | EREG | KIF1B | RELA |
| APLNR | F3 | KLF6 | RGS1 |
| AQP9 | FFAR2 | LAMP3 | RGS16 |
| ATP2A2 | FPR1 | LCK | RHOG |
| ATP2B1 | FZD5 | LCP2 | RIPK2 |
| ATP2C1 | GABBR1 | LDLR | RNF144B |
| AXL | GCH1 | LIF | ROS1 |
| BDKRB1 | GNA15 | LPAR1 | RTP4 |
| BEST1 | GNAI3 | LTA | SCARF1 |
| BST2 | GP1BA | LY6E | SCN1B |
| BTG2 | GPC3 | LYN | SELE |
| C3AR1 | GPR132 | MARCO | SELL |
| C5AR1 | GPR183 | MEFV | SELENOS |
| CALCRL | HAS2 | MEP1A | SEMA4D |
| CCL17 | HBEGF | MET | SERPINE1 |
| CCL2 | HIF1A | MMP14 | SGMS2 |
| CCL20 | HPN | MSR1 | SLAMF1 |
| CCL22 | HRH1 | MXD1 | SLC11A2 |
| CCL24 | ICAM1 | MYC | SLC1A2 |
| CCL5 | ICAM4 | NAMPT | SLC28A2 |
| CCL7 | ICOSLG | NDP | SLC31A1 |
| CCR7 | IFITM1 | NFKB1 | SLC31A2 |
| CCRL2 | IFNAR1 | NFKBIA | SLC4A4 |
| CD14 | IFNGR2 | NLRP3 | SLC7A1 |
| CD40 | IL10 | NMI | SLC7A2 |
| CD48 | IL10RA | NMUR1 | SPHK1 |
| CD55 | IL12B | NOD2 | SRI |
| CD69 | IL15 | NPFFR2 | STAB1 |
| CD70 | IL15RA | OLR1 | TACR1 |
| CD82 | IL18 | OPRK1 | TACR3 |
| CDKN1A | IL18R1 | OSM | TAPBP |
| CHST2 | IL18RAP | OSMR | TIMP1 |
| CLEC5A | IL1A | P2RX4 | TLR1 |
| CMKLR1 | IL1B | P2RX7 | TLR2 |
| CSF1 | IL1R1 | P2RY2 | TLR3 |
| CSF3 | IL2RB | PCDH7 | TNFAIP6 |
| CSF3R | IL4R | PDE4B | TNFRSF1B |
| CX3CL1 | IL6 | PDPN | TNFRSF9 |
| CXCL10 | IL7R | PIK3R5 | TNFSF10 |
| CXCL11 | CXCL8 | PLAUR | TNFSF15 |
| CXCL6 | INHBA | PROK2 | TNFSF9 |
| CXCL9 | IRAK2 | PSEN1 | TPBG |
| CXCR6 | IRF1 | PTAFR | VIP |
