# Supplementary material for: A novel inflammatory signature for evaluating immune microenvironment status in soft tissue sarcoma
Source: Front Oncol. 2022 Oct 13;12:990670. doi: 10.3389/fonc.2022.990670 (PMC9609423; doi:10.3389/fonc.2022.990670)
Supplement: Supplementary file 3 [file Table_2.docx]

| Supplementary table 2. Forty-seven known immune checkpoint-associated genes | | | |
| --- | --- | --- | --- |
| IDO1 | CD86 | CD244 | VTCN1 |
| LAG3 | PDCD1 | CD274 | CD160 |
| CTLA4 | LAIR1 | HAVCR2 | CD44 |
| TNFRSF9 | TNFRSF8 | CD27 | TNFSF18 |
| ICOS | TNFSF15 | BTLA | TNFRSF18 |
| CD80 | TNFRSF14 | LGALS9 | BTNL2 |
| PDCD1LG2 | IDO2 | TMIGD2 | C10orf54 |
| TIGIT | CD276 | CD28 | CD200R1 |
| CD70 | CD40 | CD48 | TNFSF4 |
| TNFSF9 | TNFRSF4 | TNFRSF25 | CD200 |
| ICOSLG | TNFSF14 | CD40LG | NRP1 |
| KIR3DL1 | HHLA2 | ADORA2A |  |
