# Supplementary figures and images for: A novel inflammatory signature for evaluating immune microenvironment status in soft tissue sarcoma
Source: Front Oncol. 2022 Oct 13;12:990670. doi: 10.3389/fonc.2022.990670 (PMC9609423; doi:10.3389/fonc.2022.990670)

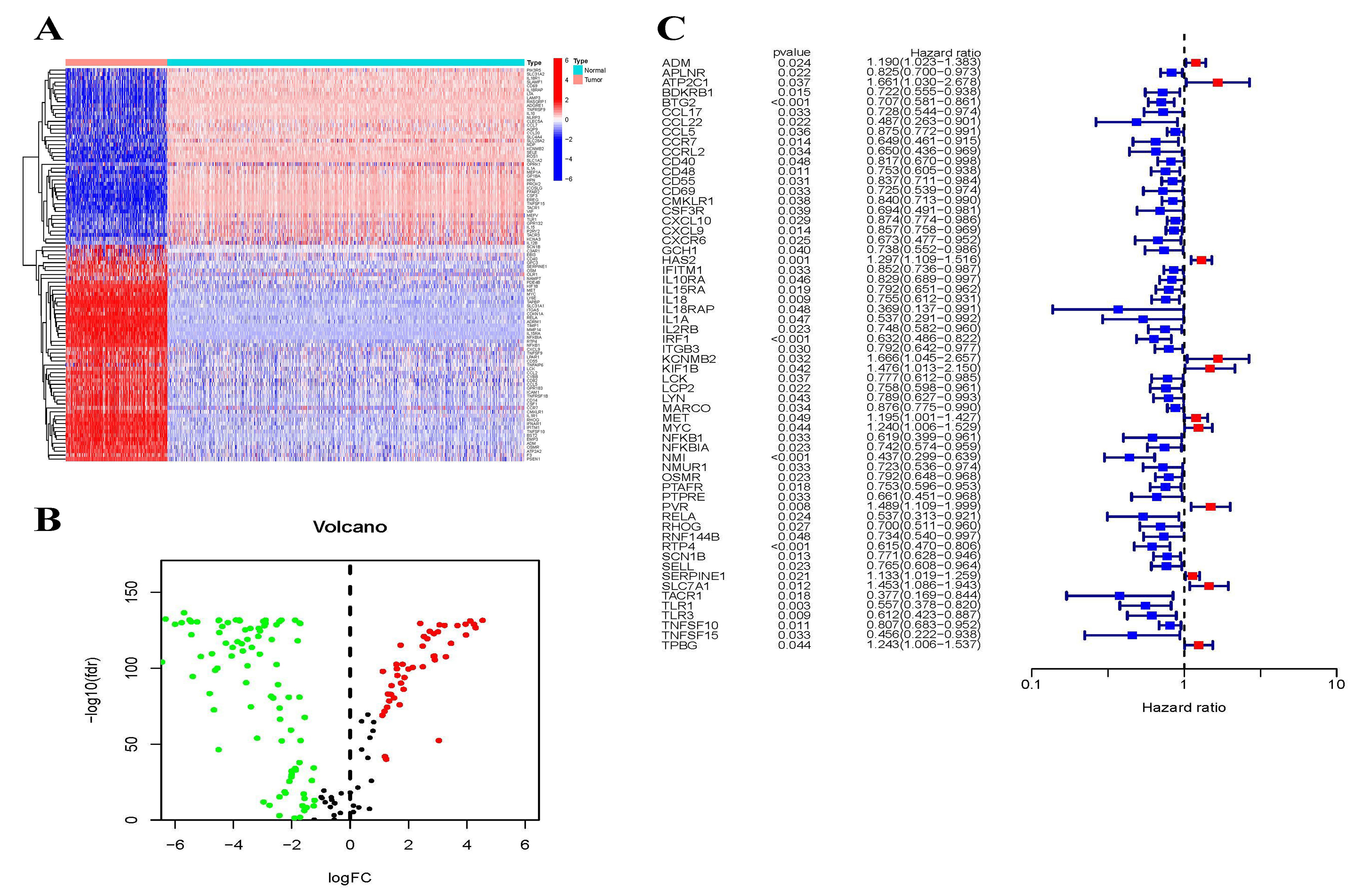

Supplement: Supplementary Figure 1 — Expression difference analysis and univariate Cox prognostic analysis. (A) Heat map of inflammatory response-related genes with significant differences. (B) Volcano plot of inflammatory response-related genes, green indicates downregulated genes, and red indicates upregulated genes. Black dots represent genes that are not differentially expressed in STS tissues and normal tissues, while green dots and red dots represent genes that are down- and up-regulated in cancer samples, respectively. (C) The forest plot shows 60 prognostic inflammatory response-related genes in terms of p-value, odds ratio (OR), and 95% confidence interval (CI). OR: odds ratio; CI: confidence interval. [file Image_1.jpeg]

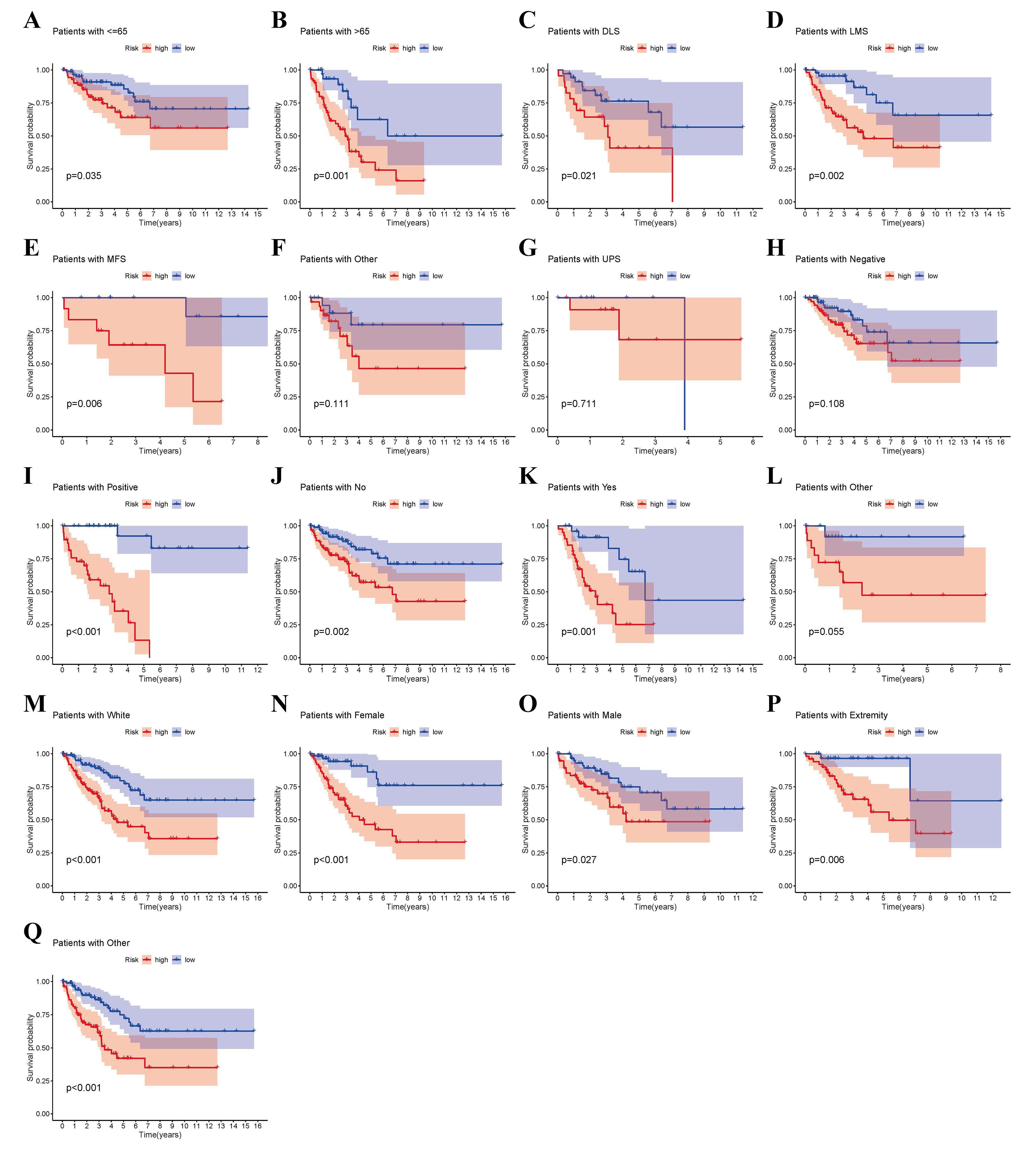

Supplement: Supplementary Figure 2 — Prognostic value of risk stratification models in multiple subgroups of soft tissue sarcoma (STS) patients. (A) STS patient with age less than or equal to 65 years old, (B) STS patient with age more than 65 years old, (C) STS patient with histological type of DLS, (D) STS patient with histological type of LMS, (E) STS patient with histological type of MFS, (F) STS patient with other histological types, (G) STS patient with histological type of UPS, (H) STS patients with negative margin status, (I) STS patients with positive margin status, (J) STS patients without metastasis, (K) STS patients with distant metastasis, (L) STS patients with other Races, (M) STS patients with White, (N) STS patients with female, (O) STS patients with male, (P) STS patients with tumor sites in the extremities, (Q) STS patients with other tumor sites. LMS: leiomyosarcoma; UPS: undifferentiated pleomorphic sarcoma; LPS: Liposarcoma; MFS: Myxofibrosarcoma; STS: soft tissue sarcomas. [file Image_2.jpeg]

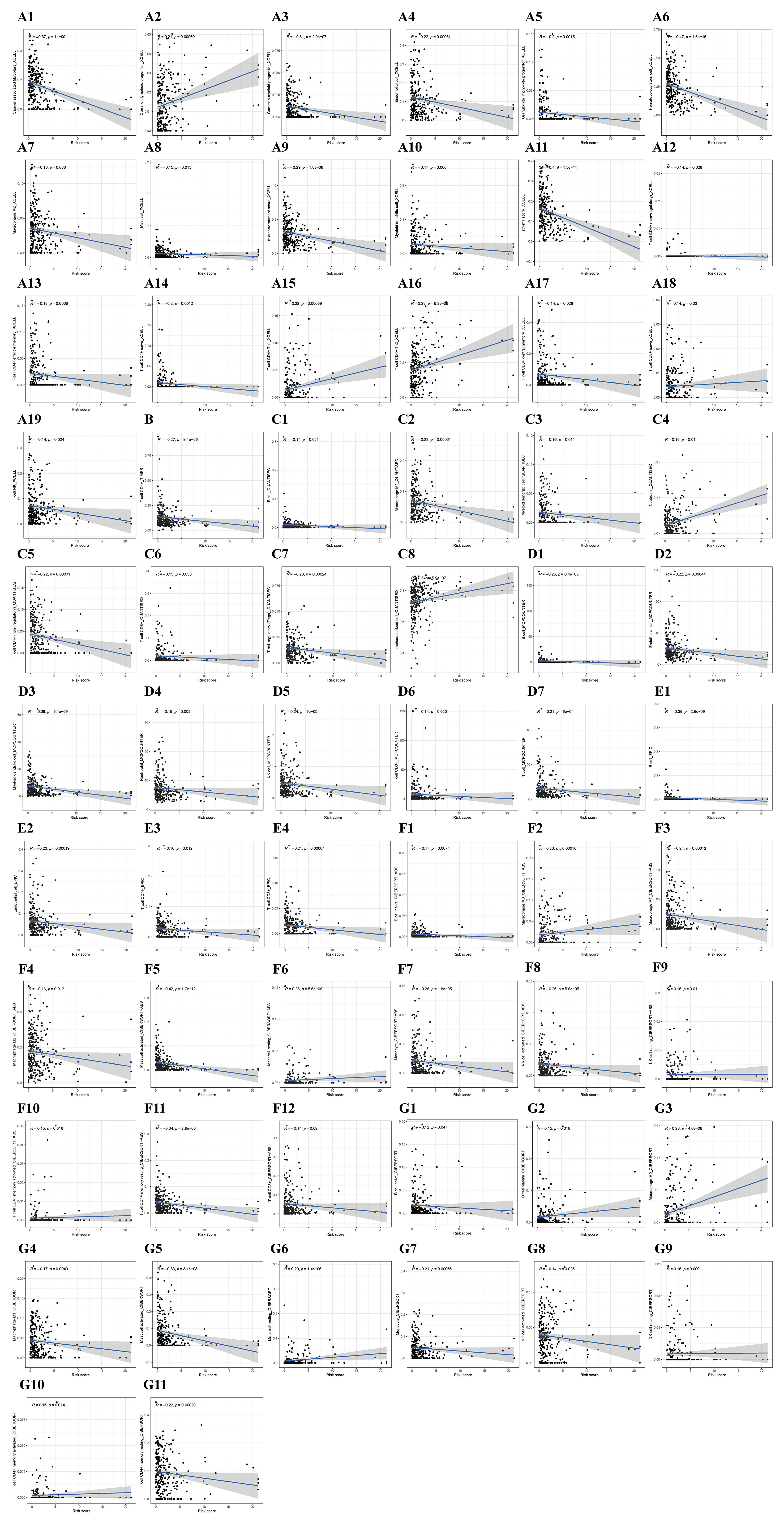

Supplement: Supplementary Figure 3 — The correlation between INFscore and immune cell infiltration. (A1–A19) Results of XCELL algorithm analysis of immune cell infiltration. (B) Results of TIMER algorithm analysis of immune cell infiltration, (C1–C8) Results of QUANTISEQ algorithm analysis of immune cell infiltration, (D1–D7) Results of MCPcounter algorithm analysis of immune cell infiltration, (E1–E4) Results of EPIC algorithm analysis of immune cell infiltration, (F1–F12) Results of CIBERSORT algorithm analysis of immune cell infiltration, (G1–G11) Results of CIBERSORT-ABS algorithm analysis of immune cell infiltration. [file Image_3.jpeg]
